# scANANSE gene regulatory network and motif analysis of single-cell clusters

**DOI:** 10.12688/f1000research.130530.1

**Published:** 2023-03-06

**Authors:** Jos G.A. Smits, Julian A. Arts, Siebren Frölich, Rebecca R. Snabel, Branco M.H. Heuts, Joost H.A. Martens, Simon J. van Heeringen, Huiqing Zhou

**Affiliations:** 1 Molecular Developmental Biology, Radboud University, Nijmegen, Gelderland, The Netherlands; 2 Molecular Biology, Radboud University, Nijmegen, Gelderland, The Netherlands; 3 Human Genetics, Radboud University Medical Centre, Nijmegen, Gelderland, The Netherlands

**Keywords:** GRN analysis, single-cell RNA-seq, single-cell ATAC-seq, Gene regulatory network, Transcription Factor

## Abstract

The recent development of single-cell techniques is essential to unravel complex biological systems. By measuring the transcriptome and the accessible genome on a single-cell level, cellular heterogeneity in a biological environment can be deciphered.

Transcription factors act as key regulators activating and repressing downstream target genes, and together they constitute gene regulatory networks that govern cell morphology and identity. Dissecting these gene regulatory networks is crucial for understanding molecular mechanisms and disease, especially within highly complex biological systems.

The gene regulatory network analysis software ANANSE and the motif enrichment software GimmeMotifs were both developed to analyse bulk datasets. We developed scANANSE, a software pipeline for gene regulatory network analysis and motif enrichment using single-cell RNA and ATAC datasets.

The scANANSE pipeline can be run from either R or Python. First, it exports data from standard single-cell objects. Next, it automatically runs multiple comparisons of cell cluster data. Finally, it imports the results back to the single-cell object, where the result can be further visualised, integrated, and interpreted. Here, we demonstrate our scANANSE pipeline on a publicly available PBMC multi-omics dataset. It identifies well-known cell type-specific hematopoietic factors. Importantly, we also demonstrated that scANANSE combined with GimmeMotifs is able to predict transcription factors with both activating and repressing roles in gene regulation.

## Introduction

Single-cell RNA-sequencing (scRNA-seq) and single-cell ATAC-sequencing (scATAC-seq), enable measurement of gene transcripts ( Islam *et al.*, 2014) and genome accessibility ( Buenrostro *et al.*, 2015) at single-cell resolution. By performing single-cell sequencing on complex biological tissues and systems, various types of cells present in the system can be identified. Furthermore, gradual changes during development and differentiation trajectories can be scrutinised. The transcriptome and accessible genome of various cell populations can be quantified, which is not obtainable using bulk analyses ( Huang, 2009; Li and Clevers, 2010). Capturing heterogeneity is vital in studying complex tissues, or while studying gradual processes such as development and differentiation, in which not all cells develop at the same rate or follow the same trajectory ( Welch, Hartemink and Prins, 2016).

One of the main drivers of differences in cellular identity and developmental processes are transcription factors (TFs). To regulate gene expression, many TFs bind the DNA directly on DNA binding motifs. These motifs are present within cis-regulatory elements (CREs), which are functionally categorised as promoters, enhancers, or insulators ( Lambert *et al.*, 2018; Chen and Pugh, 2021). These cis-regulatory elements (CREs) can be used to scan for binding motifs. However, motif enrichment does not take into account the target of CREs, the nearby genes. To better predict the impact and importance of TFs, modelling gene regulatory networks (GRNs) is preferable.

By combining (differential) gene expression, genome accessibility, and motif enrichment, with the nearby location of target genes, it is possible to generate a directed GRN. Software to predict GRNs have been actively developed since the emergence of next-generation sequencing ( Mercatelli *et al.*, 2020). The addition of genome accessibility data and incorporation of long-range CREs is a successful method to model directed-GRNs ( Xu *et al.*, 2021; González-Blas *et al.*, 2022; Kamal *et al.*, 2022). Since both scRNA-seq and scATAC-seq are available, performing directed GRN analysis can now be applied to single-cell datasets.

There are multiple single-cell-based GRN tools available, capable of combining scRNA-seq and scATAC-seq data ( Kamimoto, Hoffmann and Morris, 2020; Fleck *et al.*, 2021; González-Blas *et al.*, 2022; Kartha *et al.*, 2022). However, since single-cell data contains shallow coverage per cell and one of the main challenges these tools face is using this sparse data. Furthermore, since these tools are specifically designed for single-cell data, making comparisons of their results with available bulk datasets is challenging.

In contrast, using single-cell data from clusters as pseudo-bulk can be used relatively straightforwardly as input for many GRN tools available. To identify key TFs using GRN approaches, we previously developed the gene regulatory network analysis software ANANSE ( Xu *et al.*, 2021). ANANSE has multiple advantages: it incorporates CRE signal in 100kb windows, contains extensive TF binding models trained on the REMAP database, and can analyse data on all vertebrate species and even on non-vertebrate species with some additional steps. Theoretically, ANANSE could be run on single-cell pseudo-bulk data; however, the steps involved in generating data per cluster and running all the needed pairwise comparisons are labour-intensive and non-intuitive, while they require extensive bio-informatic skills.

Here, to enable ANANSE single-cell cluster analysis, we have developed an analysis pipeline called single-cell ANANSE (scANANSE). This pipeline consists of newly developed packages to export data from single-cell objects, either Seurat objects using the R implementation (AnanseSeurat), or from Scanpy objects using the Python implementation (AnanseScanpy). Next, an automated snakemake pipeline of ANANSE facilitates the GRN modelling. In parallel, it integrates motif enrichment analysis using GimmeMotifs ( van Heeringen and Veenstra, 2011; Bruse and Heeringen, 2018). This addition is used to identify TFs with repressive properties, which are generally not properly predicted by ANANSE. Lastly, transcription factor influence score and motif enrichment results can be imported back into the single-cell object for downstream analysis and visualisation.

The performance of the scANANSE pipeline is demonstrated on a publicly available PBMC multi-omics dataset, as an example workflow including the installation of all software needed to run the analysis. In this PBMC case study, scANANSE uncovered many well-known activating TFs within the hematopoietic lineages. Including *CEBPD* and *SPI1* in monocytes, *EBF1* and *MEF2C* in B-cells, and *STAT4* and *LEF1* in T-cells. In addition, motif enrichment and expression correlation identify both the well known repressors *PAX5* and *STAT6* within B-cells.

## Methods

### Implementation

**Figure 1.**
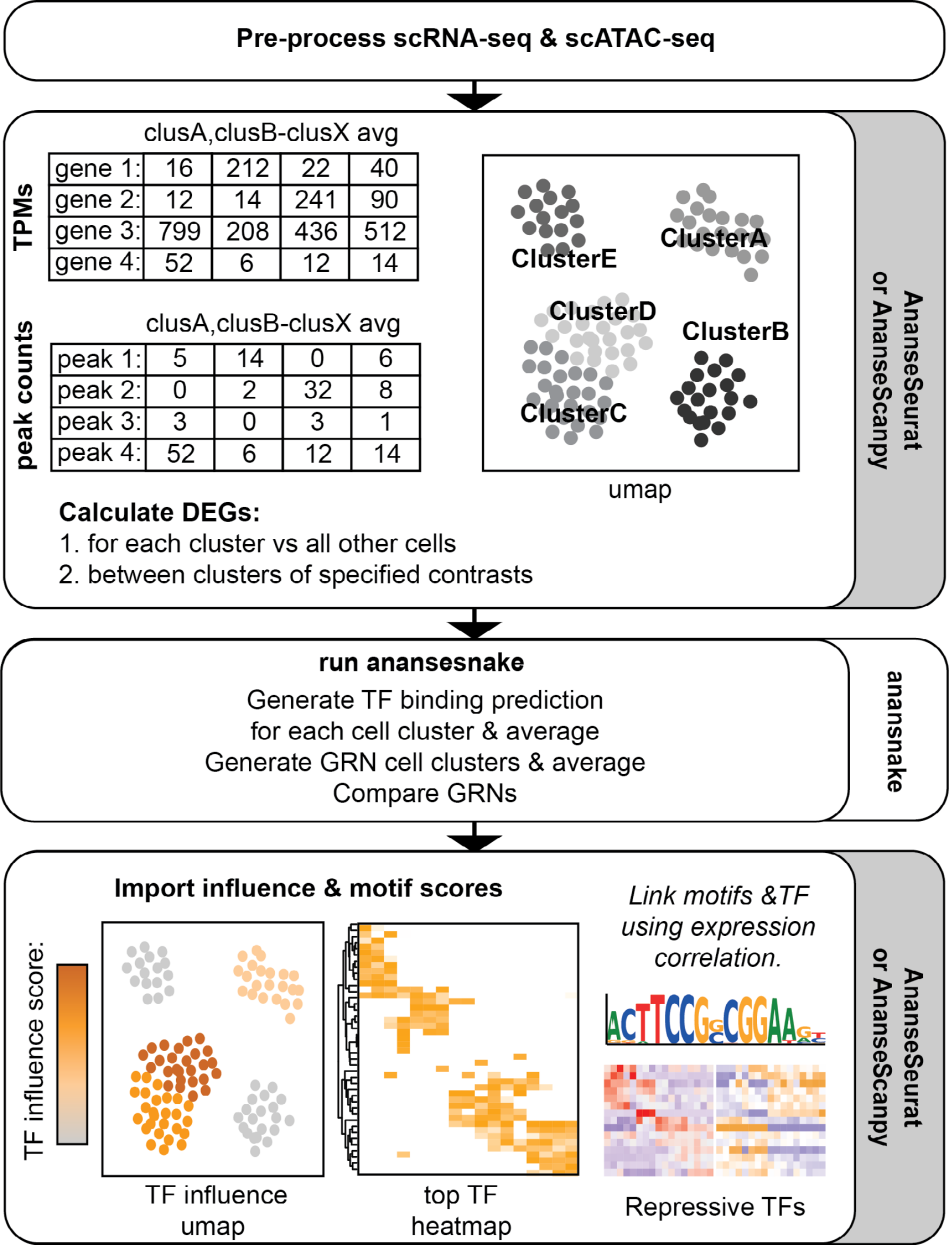
An overview of the single-cell ANANSE pipeline. After pre-processing and clustering, data is exported using either AnanseSeurat or AnanseScanpy. Next, Anansnake automatically runs ANANSE after which the influence scores and motif enrichment results with AnanseSeurat or AnanseScanpy are imported. In parallel, Anansnake runs motif enrichment analysis using gimme maelstrom, and the motif results are imported and linked to the highest correlating TFs using the single-cell object scRNA-seq data.

The scANANSE pipeline consists of two components: a package to export data from and import data towards single-cell objects, and a snakemake implementation of ANANSE called anansnake ( Figure 1). Crucial steps before running scANANSE are pre-processing, quality control, and clustering of single-cell data. For these steps, a large number of well-described workflows are available ( Zappia and Oshlack, 2018; Luecken and Theis, 2019; Baek and Lee, 2020).

scANANSE exports data from the single-cell object of choice. Transcripts Per Million (TPM), Differential Expressed Genes (DEGs) and peak counts need to be calculated based on the single-cell objects supplied. For Seurat objects in the programming language R, the R package “AnanseSeurat” was developed to perform these steps. While for Scanpy objects in the programming language Python, the Python package “AnanseScanpy” was developed.

The TPM counts, DEGs, and ATAC peak counts can be exported from one single-cell object containing both the scRNA-seq data and scATAC-seq data, or from two separate single-cell objects. In the case of two single-cell objects, these objects need to share their cluster names, e.g. by transferring anchors between separate scRNA-seq and scATAC-seq datasets ( Stuart *et al.*, 2019). As such, scRNA-seq and scATAC-seq data from multiple studies or experiments can be combined and used as input.

By default, scANANSE compares each cluster to a gene regulatory network built from the average expression and gene accessibility of all clusters. This average network is used as a common comparison to compare all clusters. These comparisons result in an average GRN ‘TF-influence’ score. This score quantifies the importance of a TF driving the differences between a specific cluster and the average of all other cell clusters. In this way, the TF influence score can be compared across multiple clusters. In addition to this general approach, more detailed direct cluster-to-cluster GRN analyses are possible.

One downside of the GRN modelling of ANANSE is the lack of prediction of repressive TFs. To counteract this blind spot of the algorithm, motif enrichment with GimmeMotifs is performed in the scANANSE pipeline. It not only performs motif enrichment but is combined with a correlation of motif-z-scores and TF expression across clusters within the single-cell object. This addition enables the ability to predict repressive TFs.

Finally, both AnanseSeurat and AnanseScanpy can be used to import the TF influence and motif enrichment scores back into your single-cell object for further visualisation and analysis. All the source code and the conda environment YAML files used to generate the results presented in this article are available in Github and Zenodo ( Arts *et al.*, 2022).

## Operation

### Minimal system requirements

A computer running UNIX, Linux, Windows Subsystem for Linux (WSL or Mac OS can run scANANSE. A minimum of 32 GB of RAM and 100 GB Of disk space is needed for a typical analysis, however, an amount of 64 GB of RAM is recommended to decrease runtime.

## Use cases: PBMC monocytes

The multi-omics dataset generated on human Peripheral blood mononuclear cells (PBMCs) publicly provided by 10× (PBMC from a Healthy Donor (v1, 150×150) Single Cell Multiome ATAC + Gene Expression Dataset by Cell Ranger ARC 2.0.0, 10× Genomics, 2022, December 20) is used as a case study. The scANANSE pipeline can also handle separate scRNA-seq and scATAC-seq objects with identical cluster names. However, within this example, scRNA-seq and scATAC-seq are part of the same single-cell object.

### Part 1: Installation and setup

The package management system Conda is installed with two environments: anansnake and scANANSE. The following folder structure is used: 
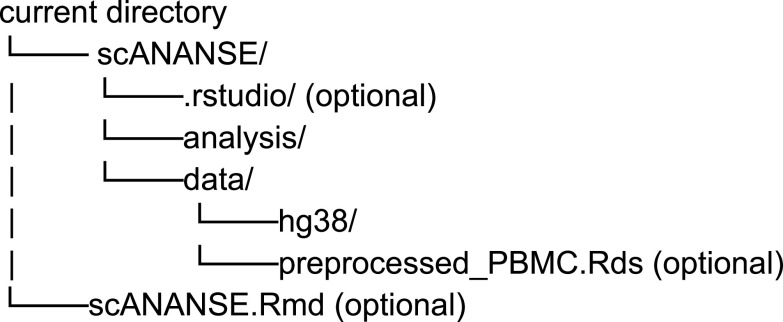


**1a. Create folders**

mkdir -p scANANSE/analysis
mkdir -p scANANSE/data


**1b. Install Conda**

The operating system and computing environment are set up as listed in the minimal system requirements. Next, Conda is installed.

# Install Conda
wget https://repo.anaconda.com/miniconda/Miniconda3-py38_4.12.0-Linux-x86_64.sh
bash Miniconda3-py38_4.12.0-Linux-x86_64.sh
rm Miniconda3-py38_4.12.0-Linux-x86_64.sh

# Configure Conda
conda config --add channels bioconda
conda config --add channels conda-forge
conda config --set channel_priority strict
conda install mamba -y


**1c. Install the anansnake Conda environment**

mamba create -n anansnake anansnake


**1d. Install the R Conda environment**

wget
https://raw.githubusercontent.com/JGASmits/AnanseSeurat/main/inst/scANANSE.yml
mamba env create -f scANANSE.yml


**1e. Install hg38**

The location where Genomepy installs genomes is set using the -g flag. Since UCSC has three annotations for hg38, the version with HGNC gene names is selected, using --UCSC-annotation. scANANSE requires HGNC gene names to run.

conda activate anansnake
genomepy install hg38 -g scANANSE/data --UCSC-annotation refGene


**1f. Install AnanseSeurat and R packages**

There are code blocks equivalent for exporting and visualising the data in python using Ananscanpy. See the extended data file “AnanseScanpy_equivalent.pdf” in the extended data ( Arts *et al.*, 2022) for these same steps but in Python. If RStudio needs to be installed on your system, see “install_Rstudio.pdf” in the extended data on Zenodo ( Arts *et al.*, 2022).

conda activate scANANSE
rstudio


From R (studio):

install.packages("AnanseSeurat")


### Part 2: Quality control and clustering of scRNA-seq and scATAC-seq data

In this example we use data from 10x pre-processed by a vignette from Signac (2022). This dataset comes with a vignette performing default quality control, clustering, and annotation from the PBMC atlas from Hao *et al.* (2021). Proper quality control and clustering are vital for all single-cell analyses for these topics, however, there already exist some excellent reviews about these topics ( Zappia and Oshlack, 2018; Luecken and Theis, 2019; Baek and Lee, 2020).

**2a. Download the raw data (optional)**

cd scANANSE/data

wget
https://zenodo.org/record/7575107/files/pbmc_granulocyte_sorted_10k_filtered_feature_bc_matrix.h5
wget
https://zenodo.org/record/7575107/files/pbmc_granulocyte_sorted_10k_atac_fragments.tsv.gz
wget
https://zenodo.org/record/7575107/files/pbmc_granulocyte_sorted_10k_atac_fragments.tsv.gz.tbi
wget
https://zenodo.org/record/7575107/files/pbmc_multimodal.h5seurat

cd ../..


**2b. Pre-process single-cell data (optional)**

An R Markdown file with all subsequent steps in R, including the pre-processing is available and can be downloaded.

wget
https://raw.githubusercontent.com/JGASmits/AnanseSeurat/main/inst/scANANSE.Rmd -O scANANSE/scANANSE.Rmd


The pre-processing analysis follows the Signac multi-omics vignette (‘ Signac’, 2022)

The QC steps can be skipped by downloading the processed Rds file.

wget
https://zenodo.org/record/7575107/files/preprocessed_PBMC.Rds


Alternatively, the processed h5ad objects for AnanseScanpy can be downloaded.

wget
https://zenodo.org/record/7575107/files/rna_PBMC.h5ad -O scANANSE/rna_PBMC.h5ad
wget
https://zenodo.org/record/7575107/files/atac_PBMC.h5ad -O scANANSE/atac_PBMC.h5ad


### Part 3: Export single-cell cluster data

**3a. Export cluster CPM, ATAC peak counts, and RNA-seq Counts**

For the ATAC-seq data, a matrix containing the counts per peak per cluster is generated. For RNA-seq, CPM equivalent values are needed. Since the data is UMI normalised, CPM is already equivalent to regular depth normalised data ( Phipson, Zappia and Oshlack, 2017). By default, scANANSE compares all clusters to a network based on the average values of all clusters. Additional comparisons can be specified, in this case, B-naive and B-memory cells were also specified to compare directly to each other.

conda activate scANANSE
rstudio




# Load the required R libraries
library(Seurat)
library(SeuratDisk)
library(stringr)
library(ComplexHeatmap)
library(circlize)
library(ggplot2)
library(AnanseSeurat)
library(SeuratDisk)

# Load pre-processed seurat object RDS file
rds_file <- './scANANSE/preprocessed_PDMC.Rds'
pbmc <- readRDS(rds_file)
export_CPM_scANANSE(pbmc,
 in_cells <- 25,
 output_dir ='./scANANSE/analysis',
 cluster_id = 'predicted.id',
 RNA_count_assay = 'RNA')

export_ATAC_scANANSE(pbmc,
 min_cells <- 25,
 output_dir ='./scANANSE/analysis',
 cluster_id = 'predicted.id',
 ATAC_peak_assay= 'peaks')

# Specify additional contrasts:
contrasts <- c('B-naive_B-memory',
         'B-memory_B-naive')
config_scANANSE(pbmc,
 min_cells <- 25,
 output_dir ='./scANANSE/analysis',
 cluster_id = 'predicted.id',
 additional_contrasts = contrasts)

DEGS_scANANSE(pbmc,
 min_cells <- 25,
 output_dir ='./scANANSE/analysis',
 cluster_id = 'predicted.id',
 additional_contrasts = contrasts)


**3b. File examples**

**Table 1.** TPM file data. Example of values and layout of the TPM.tsv file generated by the export_CPM_scANANSE() function.

	CD4-Naive	CD4-TCM	average
MIR1302-2HG	0	0	0
FAM138A	0	0	0
OR4F5	0	0	0
AL627309.1	236	1.007	1.118

**Table 2.** Peak counts file data. Example of values and layout of the Peak_Counts.tsv file generated by the export_ATAC_scANANSE() function.

	CD4-Naive	CD4-TCM	Average
chr1:10032-10322	5	22	5
chr1:180709-181030	6	20	5
chr1:181296-181600	10	12	5
chr1:191304-191914	7	12	5

**Table 3.** Marker gene file data. Example of values and layout of the hg38_cluster_average.diffexp.tsv file generated by the DEGS_scANANSE() function.

	log2FoldChange	padj
*RTKN2*	1.595.097.268	0
*FOXP3*	630.731.206	0
*IKZF2*	1.933.892.393	2.26E-241
*IL2RA*	1.372.494.589	7.02E-241

### Part 4: Anansnake

Next, snakemake is run from within a screen session. This takes approximately 3 hours per cluster plus 2 hours for motif enrichment analysis, but this is also highly dependent on computer speed. With less than 64 GB of RAM available, we recommend downscaling the core number to a maximum of 6 cores. With more RAM and more cores available the core count should be increased to reduce analysis time.

Additionally, it is possible to add extra samples and/or networks to the anansnake run. This enables including other samples and other networks in your comparisons. When performing additional anansnake comparisons please go through the anansnake documentation in detail.

screen
conda activate anansnake

# Update the timestamps so snakemake doesn't try to regenerate the DEG files if you make changes to the config or sample file
for DEGfile in scANANSE/analysis/deseq2/*;do touch -m $DEGfile;done

anansnake \
--configfile scANANSE/analysis/config.yaml \
--resources mem_mb=48_000 --cores 12


### Part 5: Import and visualise ANANSE results

**5a. Import the ANANSE results**

After running ANANSE with anansnake, the influence output is imported back into the single-cell object.

conda activate scANANSE
rstudio




pbmc <- import_seurat_scANANSE(
 pbmc,
 cluster_id = 'predicted.id',
 anansnake_inf_dir = "./scANANSE/analysis/influence"
)

# export the data per cluster from the single-cell object
TF_influence <- per_cluster_df(pbmc,
 assay = 'influence',
 cluster_id = 'predicted.id')

head(TF_influence)


**Table 4.** Influence table. Example of the influence data frame generated by per_cluster_df(assay = ‘influence’).

	B-intermediate	B-memory	B-naive	CD14-Mono	CD16-Mono
*EBF1*	1	1	1	0	0
*SPIB*	0.9375	0.913043	0	0	0
*REL*	0.875	0.391304	0.947368	0.818182	0.943396
*TCF4*	0.8125	0.695652	0.736842	0	0
*BACH2*	0.75	0.391304	0.894737	0	0

**5b. Top five influential TFs per cluster**

Next, the top five TFs per cluster are identified from the influence table.

TF_influence$TF <- rownames(TF_influence)
TF_long <- reshape2::melt(TF_influence, id.vars = 'TF')
colnames(TF_long) <- c('TF','cluster', 'influence')
TF_influence$TF <- NULL
TF_long <- TF_long[order(TF_long$influence, decreasing = TRUE), ]

# get the top n TFs per cluster
topTF <- Reduce(rbind,
 by(TF_long,
  TF_long["cluster"],
  head,
  n = 5))# Top N highest TFs by cluster

top_TFs <- unique(topTF$TF)

TF_table <- topTF %>%
 dplyr::group_by(cluster) %>%
 dplyr::mutate('TopTFs' = paste0(TF, collapse = " "))

unique(TF_table[,c('cluster','TopTFs')])


**Table 5.** Top five TF influence scores per cell type. Referenced TFs in the text are in bold and highlighted.

Cluster	Cluster type:	Top TFs				
CD14-Mono	monocytes	*BACH1*	***CEBPD***	*FOXO3*	*JUN*	*RBPJ*
CD16-Mono		*MAFB*	*NR4A1*	*RARA*	*RXRA*	***SPI1***
pDC	Dendritic cells	*BCL11A*	*CUX2*	***IRF4***	*MYBL2*	*SPIB*
cDC2		*BCL11A*	*BHLHE40*	*ETS2*	*RUNX2*	*SPI1*
HSPC	progenitor cells	***ERG***	*ETV6*	***GATA2***	***MEIS1***	*MYB*
B-intermediate	B cells	*BACH2*	***EBF1***	***MEF2C***	*REL*	*SPIB*
B-memory		*BCL11A*	***EBF1***	***MEF2C***	***PAX5***	*REL*
B-naive		*BACH2*	*BCL11A*	***EBF1***	***FOXO1***	*REL*
CD4-Naive	CD4 T-cells	*BACH2*	***FOXO1***	***FOXP1***	***LEF1***	*TCF7*
CD4-TCM		***GATA3***	***LEF1***	*MAF*	*RORA*	*TCF7*
CD4-TEM		*MAF*	*PBX4*	*RORA*	*STAT4*	*TCF7*
Treg		*ETS1*	***GATA3***	***LEF1***	*PRDM1*	*RORA*
CD8-Naive	CD8 T-cells	*BACH2*	***FOXO1***	***FOXP1***	***LEF1***	*TCF7*
CD8-TCM		***GATA3***	*KLF9*	*NR3C2*	*RUNX3*	***STAT4***
CD8-TEM	NK-cells	***EOMES***	*MYBL1*	*RORA*	*RUNX3*	*TBX21*
gdT	other T-cells	*IKZF2*	*MYBL1*	*RORA*	*RUNX3*	***STAT4***
MAIT		***EOMES***	*IKZF2*	*RORA*	*RORC*	***STAT4***
NK	NK-cells	*RORA*	*RUNX3*	***STAT4***	*TBX21*	*XBP1*

**5c. Heatmap of most influential TFs**

**Figure 2.**
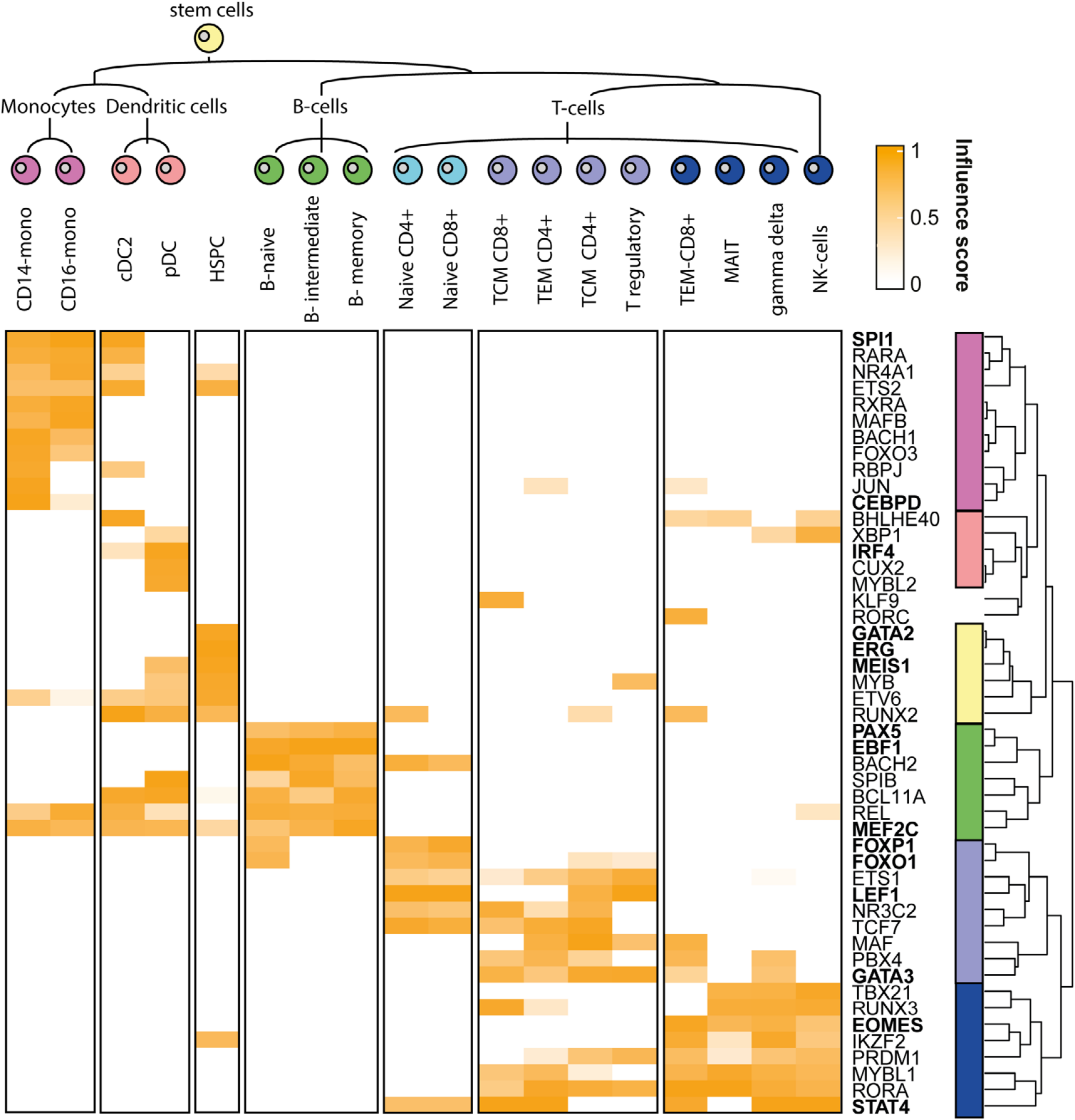
Heatmap of the influence scores. This heatmap depicts the influence scores of the top five highest influential TFs per cluster. Referenced TFs in the text are in bold.

An overview of the top TF and their various influences in the various clusters is visualised by a heatmap. The column and row dendrogram are manually swapped where appropriate resulting in the final TF influence heatmap (see Figure 2).

col_fun = circlize::colorRamp2(c(0, 1), c("white", "orange"))
mat <- as.matrix(TF_influence[rownames(TF_influence) %in% top_TFs,])

pdf('./scANANSE/analysis/ANANSE_Heatmap.pdf',width=16,height=8,paper='special')
ComplexHeatmap::Heatmap(mat, col = col_fun)
dev.off()


By using scANANSE, a large number of well-known hematopoiesis hallmark TFs is identified (see Figure 2 and Table 5). This demonstrates the ability of scANANSE to identify important transcription factors from single-cell data. Some well-known examples are:

Monocytes TFs include, *SPI1 which* is well known to regulate human monocyte differentiation towards dendritic cells and is identified in both monocytes and dendritic cells ( Rosa *et al.*, 2007; Novershtern *et al.*, 2011; Zhu *et al.*, 2012). While the *CEBP* gene family, including the identified *CEBPD*, is vital for the transduction of B-cells into macrophages ( Bussmann *et al.*, 2009).

Dendritic cell TFs including *IRF4* ( Tamura *et al.*, 2005) were identified as driving Interferon producing pDCs ( Siegal *et al.*, 1999).

Hematopoietic stem cell TFs include *GATA2* ( Menendez-Gonzalez *et al.*, 2019, p. 2), *ERG* ( Knudsen *et al.*, 2015) and *MEIS1* ( Novershtern *et al.*, 2011; Ariki *et al.*, 2014). All these factors are all well-known regulators of hematopoietic stem cell identity.

B-cell TFs include *EBF1* and *MEF2C.* Both are well-known to drive the B-cell lineage ( Kong *et al.*, 2016; Bullerwell *et al.*, 2021, p. 1), while *PAX5* is another well-known B-cell fate driving factor ( Enver, 1999, p. 5; Medvedovic *et al.*, 2011, p. 5). In particular, *PAX5* is an intriguing finding since it is not only well known to promote B-cell genes, but also to repress non-B-cell lineage genes ( Boller and Grosschedl, 2014). This repressive property is however not included in the ANANSE analysis. And its prediction is likely attributed to the smaller effect of gene activation PAX6 has on specific target genes.

T-cell TFs include both *GATA3* and *LEF1*, which are crucial for specifying the T-cell fate ( Novershtern *et al.*, 2011). Furthermore, more specific to naive T-cells, *FOXO1 *( Kerdiles *et al.*, 2009, p. 1) and *FOXP1* ( Feng *et al.*, 2010) are known to maintain naive T-cell quiescence.

Differentiated T-cell TFs include the well-known *STAT4* ( Novershtern *et al.*, 2011; Suarez-Ramirez *et al.*, 2014), and for both CD8+ T-cells and NK cells the well-known TF *EOMES* ( Shimizu *et al.*, 2019) are identified.

**5d. Visualise TF expression and influence on a UMAP**

**Figure 3.**
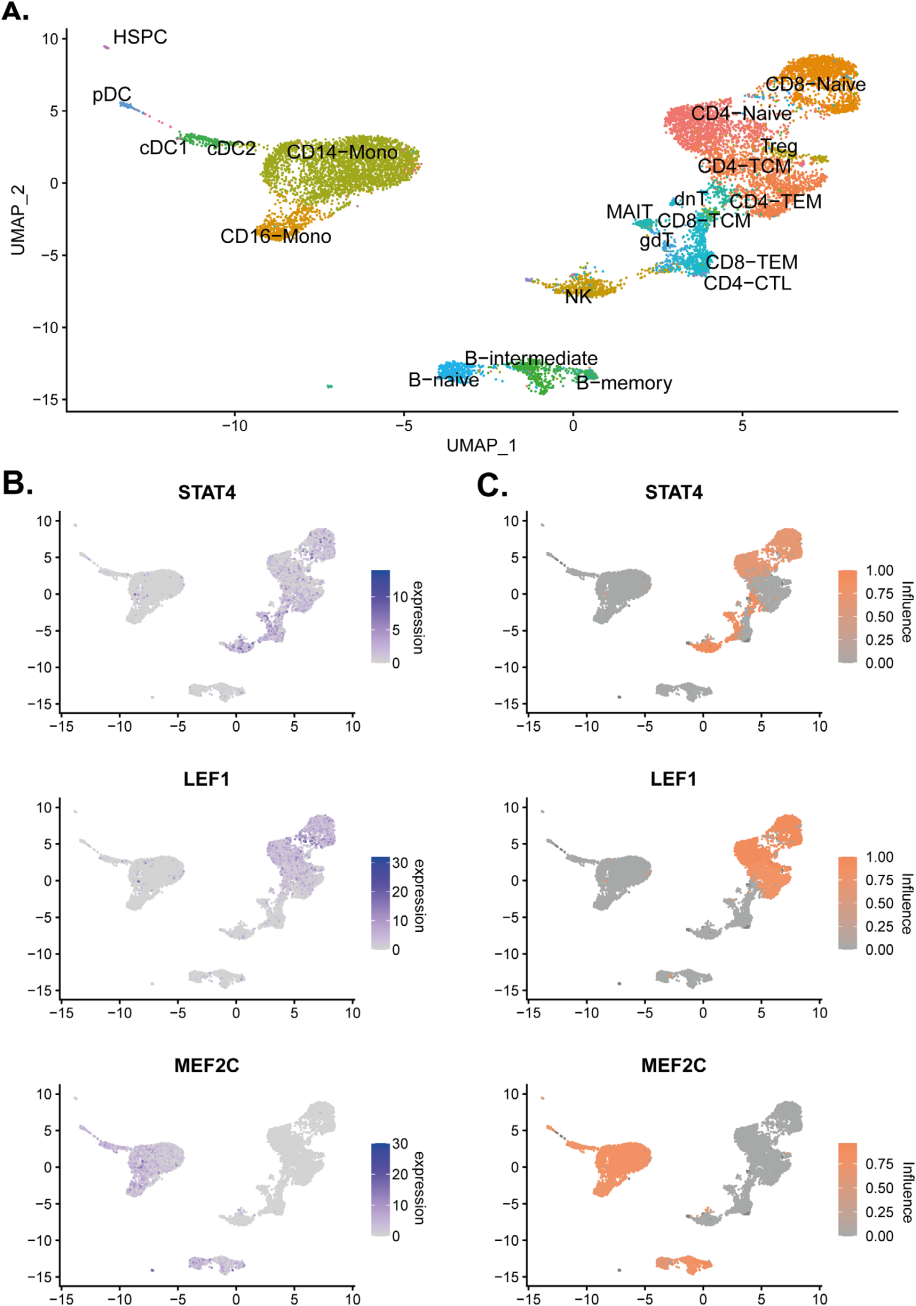
Expression and influence visualisation upon the UMAP. (A) UMAP of the PBMC single-cell object with the cell identities labelled. (B) Normalised expression values of *STAT4*, *LEF1*, and *MEF2C* on the single-cell object. (C) Influence scores of *STAT4*, *LEF1*, and *MEF2C* on the single-cell object.

The presence of the influence scores enabled clear visualisation of the influence and expression of specific TFs across the dataset. As an example, three TFs are visualised with a wide variety of influence and expression across clusters (see Figure 3).

highlight_TF1 <- c('STAT4','LEF1','MEF2C')

Annotated_plot <- DimPlot(pbmc,
 label = T,
 repel = TRUE,
 reduction = "umap")+ NoLegend()

DefaultAssay(object = pbmc) <- "RNA"
plot_expression <- FeaturePlot(pbmc,
 features = highlight_TF1,
 ncol = 1)

DefaultAssay(object = pbmc) <- "influence"
plot_ANANSE <- FeaturePlot(pbmc,
 ncol = 1,
 features = highlight_TF1,
 cols = c("darkgrey", "#fc8d59"))

pdf('./scANANSE/analysis/ANANSE_highlight.pdf',width=10,height=10,paper='special')
print(Annotated_plot)
print(plot_expression|plot_ANANSE)
dev.off()


### Part 6: Specific cluster comparison

Although all B-cell clusters were relatively similar when compared to the average network, it is possible to directly compare both clusters. This uncovers TFs driving more subtle differences between the cell types. This direct cluster-to-cluster comparison is performed by adding the two clusters in part 3 as an additional contrast.

**Figure 4.**
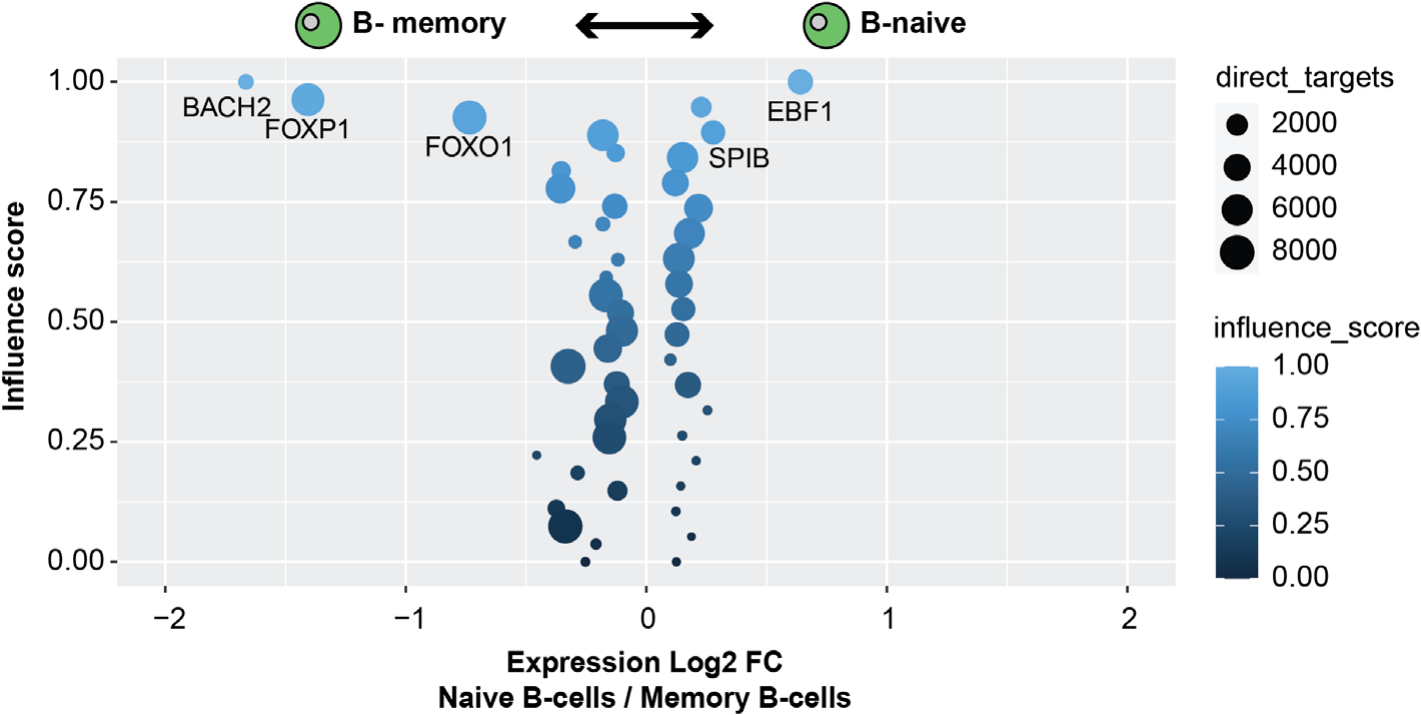
ANANSE can perform direct cluster-to-cluster comparisons. The TF influence scores of TFs comparing Naive B-cells and Memory B-cells; higher influence of factors with negative fold changes are more important within memory B-cells; higher influence of factors with positive fold changes are more important in Memory B-cells. Circle size correlates with the number of direct target genes. Gene expression log2 fold change between Naive B-cells and memory B-cells on the X axis.

When comparing Naive B-cells and Memory B-cells, *FOXP1* and *BACH2* were identified as important factors driving Memory B-cell maturation compared to naive B-cells. This is in line with previous publications ( Itoh-Nakadai *et al.*, 2014; Patzelt *et al.*, 2018). Furthermore, EBF1 and SPIB were identified as driving Naive B-cells, this is also in line with previous research ( Schmidlin *et al.*, 2008; Györy *et al.*, 2012). Thus, these results illustrate the possibility of running comparisons on similar clusters within single-cell datasets to further identify TF networks that define cell types ( Figure 4).

MemoryInfluence <- read.table(
 './scANANSE/analysis/influence/anansesnake_B-memory_B-naive.tsv',
 header = T)
NaiveInfluence <- read.table(
 './scANANSE/analysis/influence/anansesnake_B-naive_B-memory.tsv',
 header = T)

NaiveInfluence$factor_fc <- NaiveInfluence$factor_fc* -1
B_comparison <- rbind(NaiveInfluence,MemoryInfluence)

ggplot(B_comparison, aes(factor_fc,influence_score)) +
 geom_point(aes(size = direct_targets, colour = influence_score)) +
 xlim(-2,2)+
 geom_text(
  aes(
   label=ifelse(factor_fc > 0.26|factor_fc < -0.5,as.character(factor),""),
  hjust = 0.5,
  vjust = 2
))


### Optional part: Motif enrichment for predicting repressive factors

Since ANANSE's assumptions for GRN modelling are not valid for repressive factors, one limitation is the inability to reliably predict repressive TFs. Motif enrichment can be used for identifying motifs with reduced accessibility, however due to the lack of a one-on-one link of motifs and TFs, and the difference of these interactions between tissues, it is tricky to reliably link motifs with their most relevant factors in the cell type of interest.

However, with single-cell cluster data, it is possible to link motifs and TFs based on motif and expression correlation across multiple clusters. This approach does enable scANANSE to identify potential repressive factors. It is however a step down from the GRN modelling approach, but for identifying potential repressive factors it is an easy step to incorporate, which we therefore choose to include.

We will first incorporate the enrichment result after running anansesnake.

**Import motif enrichment scores**

pbmc <- import_seurat_maelstrom(pbmc,
 cluster_id = 'predicted.id',
 maelstrom_file = './scANANSE/analysis/maelstrom/final.out.txt')

# export the data per cluster from the single-cell object
motif_scores <- per_cluster_df(pbmc,
 assay = 'maelstrom',
 cluster_id = 'predicted.id')

head(motif_scores)


**Table 6.** Motif score table. Example of the Motif score data frame generated by per_cluster_df(assay = ‘maelstrom’).

	CD4-Naive	CD4-TCM	CD8-Naive	CD16-Mono	NK
GM.5.0.GATA.0013	2.615203	1.610542	4.671053	-5.09854	2.947372
GM.5.0.C2H2-ZF.0188	-0.54753	-0.40019	1.878645	0.401215	1.746373
GM.5.0.Nuclear-receptor.0109	-1.65902	-2.74995	-1.7461	2.672638	-0.32766
GM.5.0.Forkhead.0058	0.629503	0.139509	-0.17629	0.621033	1.049713

**Link TFs to motifs based on their correlation coefficient**

The enriched motifs are linked to TFs based on the non-redundant motif-TF database generated by GimmeMotifs. A correlation score is calculated between the motif-z-scores and TF expression values. When multiple TFs map to the same motif of interest, the TF with the highest absolute correlation is linked to this motif. After linking all motifs, one TF can be linked to multiple motifs. In that case, there are multiple options for selecting the most relevant motif.

First of all, it is possible to take the mean motif score, secondly by selecting the motif with the most variable signal, or thirdly by selecting the motif with the highest absolute correlation between enrichment and expression. Here we use the motifs with the highest correlation to the expression.

Finally, two assays are added to the single-cell object, one consisting of a positive correlation with linked motifs, which indicates a TF promoting genome accessibility, and one assay consisting of a negative correlation with linked motifs, which indicates TFs repressing genome accessibility. A TF can be present in both assays when it is linked both with a motif with a positive correlation and a motif with a negative correlation.

pbmc <- Maelstrom_Motif2TF(pbmc,
 cluster_id = 'predicted.id',
 maelstrom_dir = './scANANSE/analysis/maelstrom/',
 RNA_expression_assay = "SCT",
 output_dir ='./scANANSE/analysis',
 expr_tresh = 10,
 cor_tresh = 0.3,
 combine_motifs = 'max_cor')


**Visualise TF expression and motif enrichment**

**Figure 5.**
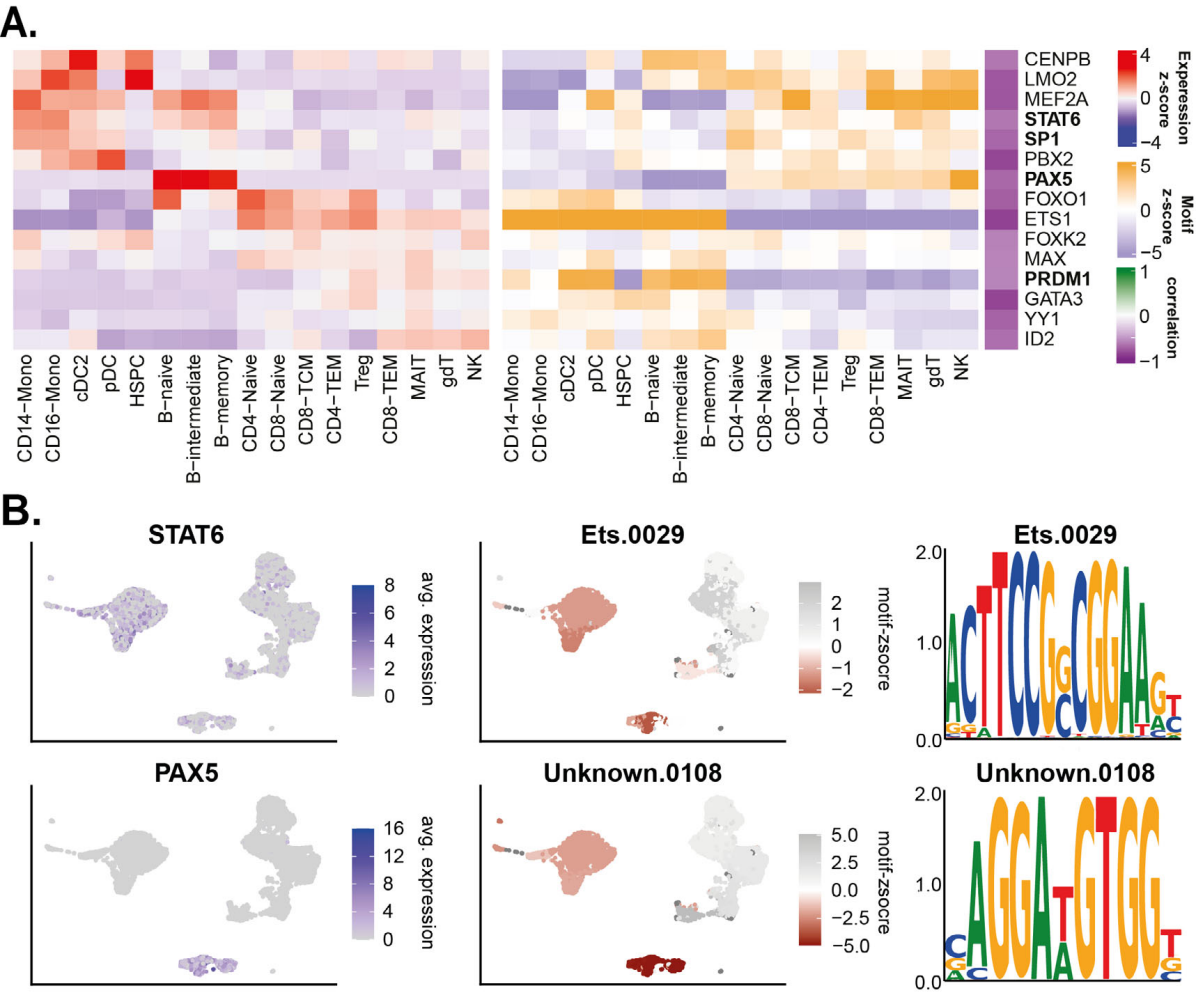
Motif enrichment repressive TFs. (A) Heatmap of top negatively correlating motifs & TFs. (B) UMAP example of anti-correlation factors PAX5 and STAT6.

Next, the top TFs of with a negative correlation were visualised as a heatmap ( Figure 5A).

col_fun <- circlize::colorRamp2(c(-5,0,5), c('#998ec3','white','#f1a340'))
col_fun_cor <- circlize::colorRamp2(c(-1,0,1), c('#7b3294','#f7f7f7','#008837'))

for (regtype in c('TFcor','TFanticor')){
 top_TFs <- head(pbmc@assays[[regtype]][[]],15)
 mat <- per_cluster_df(pbmc, assay = regtype, cluster_id = 'predicted.id')
 mat <- as.matrix(mat[rownames(mat) %in% rownames(top_TFs),])

 #get TF expression matrix
 exp_mat <- AverageExpression(pbmc,assay='SCT',
  slot = 'data',
  features = rownames(top_TFs),
  group.by = 'predicted.id')[[1]]

 exp_mat <- exp_mat[,colnames(exp_mat)]
 exp_mat <- as.matrix(t(scale(t(exp_mat))))
 #get correlation score
 row_ha = rowAnnotation(correlation = top_TFs$cor, col = list(correlation = col_fun_cor))
 print(Heatmap(exp_mat[,cluster_order], cluster_columns = F) + Heatmap(mat[,cluster_order], col = col_fun, cluster_columns = F, right_annotation = row_ha))
 }


This identified multiple repressive hallmark TFs ( Figure 5A). Examples and well known important repressors driving hematopoiesis include *PAX5* ( Souabni *et al.*, 2002, p. 1), *STAT6* ( Czimmerer *et al.*, 2018), *ID2* ( Ji *et al.*, 2008), and *PRDM*1 ( Chan *et al.*, 2009, p. 1; Nadeau and Martins, 2022).

TF_list <- c('PAX5','STAT6')
Factor_Motif_Plot(pbmc, TF_list, assay_maelstrom = 'MotifTFanticor', logo_dir = './scANANSE/analysis/maelstrom/logos/')


## Conclusions

Here we demonstrate that scANANSE is able to decipher the gene regulatory networks driving the identity of single-cell clusters. This enables the identification of TFs that drive the cellular identity of single-cell clusters of scRNA-seq and scATAC-seq datasets.

Currently, there are multiple other tools available and under development for performing GRN analysis using a combination of scRNA-seq and scATAC-seq data. Examples include software such as SCENIC+( González-Blas *et al.*, 2022), Pando ( Fleck *et al.*, 2021), CellOracle ( Kamimoto, Hoffmann and Morris, 2020) and FigR ( Kartha *et al.*, 2022). These tools have the advantage and the challenge of calculating GRNs using individual cells. While they are not relying on clustering before GRN analysis, these tools struggle at identifying low expressed target genes and TFs since individual cells have low transcriptome coverage. Comparing and benchmarking all these single-cell GRN tools is beyond the scope of this paper, but would be an exciting addition to the field in the future.

scANANSE has some clear advantages. First of all, it has the ability to analyse single-cell data generated from all vertebrate genomes. When working with non-vertebrate data, extra steps for identifying homologous genes across phyla are required before running scANANSE. For more information on that topic, see the ANANSE documentation on the motif database. This flexibility enables GRN analysis on single-cell data from a high variety of organisms. Furthermore, due to the pseudo-bulk approach, it is possible to compare single-cell cluster gene regulatory networks against networks generated from traditional bulk sequencing data. Although the amount of publicly accessible single-cell datasets is growing, there is an even larger amount of bulk sequencing datasets available. Moreover, the possibility and flexibility of comparing GRNs from multiple sources is another advantage of scANANSE, extra care and validation is still needed when using networks from different data sources.

scANANSE makes a few assumptions that are important to note regarding the average network comparison. Using the average network as the background comparison against each cluster-specific network enables the identification of TFs driving each specific cluster. In the case of small cluster numbers, this approach is however limiting the reliability and the number of factors identified since the average network contains accessibility data from all clusters including the cluster being compared. In cases with low cluster numbers, it is therefore recommended to run scANANSE including pairwise comparisons between all the clusters.

Another limitation of the GRN modelling of ANANSE is its inability to predict repressive transcription factors, or factors with context-dependent and/or repressive properties ( Krishnakumar *et al.*, 2016; Pang and Snyder, 2020). While deciphering molecular mechanisms, the inclusion of repressive factors and factors with context-dependent purpose is highly useful ( Gaston and Jayaraman, 2003; Bauer, Buske and Bailey, 2010; Arnold *et al.*, 2013). ANANSE however uses a rank mean approach which assumes all TF target gene relations are activating, while furthermore requiring a TF to be higher expressed. These assumptions are not always applicable to TFs with repressive or context-dependent functions ( Xu *et al.*, 2021). To alleviate some of this limitation, we have integrated motif enrichment analysis from the GimmeMotifs toolkit. Combining the motif z-score with a correlation of TF expression provides a straightforward tool to link motifs to the most relevant TFs which can be repressive. However, this approach does not take into account the potential combinatorial function of TFs ( Zeitlinger, 2020) and/or missing interactions in the TF to motif database.

With scANANSE, we have implemented a robust and capable toolkit to identify key TFs important for driving cellular identity and differentiation in single-cell data. It relies on solid pseudo-bulk signals and proven bulk-GRN approaches to identify the TFs of interest.

## Data Availability

PBMC datasets used in this study were obtained from 10x Genomics ( 10x Genomics, 2021), This data is available under the terms of the Creative Commons Four (CC BY 4.0). The reference PBMC dataset used for cluster annotation was obtained from Hao et al ( Hao *et al.*, 2021). Zenodo: Datasets accompanying scANANSE ( Arts *et al.*, 2022). https://doi.org/10.5281/zenodo.7575107 This project contains the following underlying data:
•

pbmc_granulocyte_sorted_10k_atac_fragments.tsv.gz (raw datafile1 ( 10x Genomics, 2021)) •

pbmc_granulocyte_sorted_10k_atac_fragments.tsv.gz.tbi (raw datafile2 ( 10x Genomics, 2021)) •

pbmc_granulocyte_sorted_10k_filtered_feature_bc_matrix.h5 (raw datafile3 ( 10x Genomics, 2021)) •
pbmc_multimodal.h5seurat (Reference PBMC dataset used for cluster annotation from Hao *et al.* (2021)) Preprocessed single cell objects, code to install Rstudio and the python code equivalent for all the steps are available as well in Zenodo archive as extended data. This project contains the following extended data:
•
rna_PBMC.h5ad (Processed Scanpy object containing the PBMC dataset scRNAseq data after quality control clustering and annotation) •
atac_PBMC.h5ad (Processed Scanpy object containing the PBMC dataset scATACseq data after quality control clustering and annotation) •
preprocessed_PBMC.Rds (Processed Seurat object containing the PBMC dataset after quality control clustering and annotation) •
Install_Rstudio.pdf (code to install Rstudio on your machine) •
AnanseScanpy_equivalent.pdf (code of the Python equivalent of all R code present in this manuscript)

## Software availability

• AnanseSeurat is https://github.com/JGASmits/AnanseSeurat (version 1.1.0). It is furthermore downloadable available from CRAN: https://cran.r-project.org/web/packages/AnanseSeurat/index.html
• AnanseScanpy is https://github.com/Arts-of-coding/AnanseScanpy (version 1.0.0) It is furthermore available from bioconda: https://anaconda.org/bioconda/anansescanpy
• Anansnake is https://github.com/vanheeringen-lab/anansnake It is furthermore available from bioconda: https://anaconda.org/bioconda/anansnake

License: AnanseSeurat, AnanseScanpy and Anansnake are all available under an Apache License 2.0
