# Supplementary material for: scANANSE gene regulatory network and motif analysis of single-cell clusters
Source: F1000Res. 2023 Mar 6;12:243. [Version 1] doi: 10.12688/f1000research.130530.1 (PMC10728588; doi:10.12688/f1000research.130530.1)
Supplement: Peer review history

# Reviewer report: Reviewer response for version 1

**DOI:** 10.5256/f1000research.143297.r186167

**Published:** 2023-07-26

**Authors:** Marouen Ben Guebila

**Affiliations:** 1 T.H. Chan School of Public Health, Harvard University, Cambridge, Massachusetts, USA

**Summary**:

 scANANSE is a software tool for modeling cis-gene regulatory networks in single-cell data, which is a relevant question as we lack tools that can scale up to large data sets. The tool combines ANANSE and GimmeMotifs, methods previously published by the authors in a single pipeline along with plotting and clustering functions that make it straightforward for users to run analyses rapidly.

 This contribution is timely and relevant to current challenges in the field and the tutorial part is very clear and easy to follow. However, I have a few suggestions to improve readability and address a few concerns about the rationale of this approach.

**Comments**:

 “However, since single-cell data contains shallow coverage per cell and one of the main challenges these tools face is using this sparse data.”

 -> Would it be possible to rephrase this sentence to convey the meaning in a better way

 “In contrast, using single-cell data from clusters as pseudo-bulk can be used relatively straightforwardly as input for many GRN tools available.”

 -> I suggest rephrasing “relatively straightforwardly”

 Bio-informatic/bulk-GRN/directed-GRNs

 -> Hyphens are not needed here

 transcription factor and gene regulatory as TF and GRNs

 -> Abbreviations for these words were defined in the beginning so they should be used afterwards. 

 “a package to export data from and import data towards single-cell objects”

 There is a word missing or sentence construction not clear.

 Please cite R and Python in the text for example in “objects in the programming language Python”

 “One downside of the GRN modelling of ANANSE is the lack of prediction of repressive TFs.” 

 ->This is very interesting, would it be possible to provide an example or a reference to this statement. -> Why is it this case? and how does your approach-combining with motif data-helps? Generally, most TFs are activators and do so by opening the chromatin for transcription.

 “Since ANANSE's assumptions for GRN modelling are not valid for repressive factors, one limitation is the inability to reliably predict repressive TFs.”

 -> Can you please state these assumptions

 I understand that the motif infers TF motif from DEGs and then links them to candidate TFs using expression correlation. Did the authors consider using TF motifs from HT-SELEX experiments in HOCOMOCO or CIS-BP databases instead?

 In Table 3, gene names are Italic but not in table1. TFs are proteins and so it is possible to not italicize their symbols in the text and table 5 for example.

 “Although the amount of publicly accessible single-cell datasets is growing, there is an even larger amount of bulk sequencing datasets available” 

 -> This sentence needs to be connected to its main idea.

 Finally, I briefly reviewed ANANSE since scANANSE inherits parts of its strengths and weaknesses. A recent benchmark (FIgure 3A in DOI 10.15252/msb.202311627) stated that “enhancer-based network ANANSE showed very poor performance across all cell types”. I believe that this opportunity to address these comments by doing additional benchmarks and identifying the parameter regions/specific use case where scANANSE operates in its optimal regime. I think this conversation can help the community advance on these topics.

Are the conclusions about the tool and its performance adequately supported by the findings presented in the article?

Yes

Is the rationale for developing the new software tool clearly explained?

Yes

Is the description of the software tool technically sound?

Yes

Are sufficient details of the code, methods and analysis (if applicable) provided to allow replication of the software development and its use by others?

Yes

Is sufficient information provided to allow interpretation of the expected output datasets and any results generated using the tool?

Yes

Reviewer Expertise:

Bioinformatics, gene regulatory networks

I confirm that I have read this submission and believe that I have an appropriate level of expertise to confirm that it is of an acceptable scientific standard, however I have significant reservations, as outlined above.

## References

[1] : GRaNIE and GRaNPA: inference and evaluation of enhancer-mediated gene regulatory networks. *Mol Syst Biol* .2023;19(6) : 10.15252/msb.202311627 e11627 10.15252/msb.202311627 37073532 PMC10258561

# Response

**Published:** 2023-11-24

**Authors:** Jos Smits

**Affiliations:** Radboud University Nijmegen, The Netherlands

**Dear Dr. Marouen Ben Guebila,**

**Thank you for providing suggestions to further improve our manuscript. We have revised the manuscript accordingly. Next to incorporating the suggested changes we have also updated AnanseSeurat to be compatible with the recently released Seurat V5. A point-by-point response can be found bellow. Especially regarding the benchmarking question we are happy to elaborate bellow.**

***Summary*** *:*

*scANANSE is a software tool for modeling cis-gene regulatory networks in single-cell data, which is a relevant question as we lack tools that can scale up to large data sets. The tool combines ANANSE and GimmeMotifs, methods previously published by the authors in a single pipeline along with plotting and clustering functions that make it straightforward for users to run analyses rapidly.*

*This contribution is timely and relevant to current challenges in the field and the tutorial part is very clear and easy to follow. However, I have a few suggestions to improve readability and address a few concerns about the rationale of this approach.*

***Comments**:*

*“However, since single-cell data contains shallow coverage per cell and one of the main challenges these tools face is using this sparse data.”*

*-> Would it be possible to rephrase this sentence to convey the meaning in a better way*

**We rephrased the sentence into: “However, since single-cell data contains relatively low genome and/or transcriptome coverage per cell compared to bulk, one of the main challenges these tools face is using this sparse data** **”**

*“In contrast, using single-cell data from clusters as pseudo-bulk can be used relatively straightforwardly as input for many GRN tools available.”*

*-> I suggest rephrasing “relatively straightforwardly”*

**We rephrased the sentence into: “In contrast, using single-cell data from robust clusters as pseudo-bulk can be used as input for many GRN tools available**”

*Bio-informatic/bulk-GRN/directed-GRNs*

*-> Hyphens are not needed here*

**We removed the hyphens**

*transcription factor and gene regulatory as TF and GRNs*

*-> Abbreviations for these words were defined in the beginning so they should be used afterwards.*

**We fixed inconsistent abbreviations**

*“a package to export data from and import data towards single-cell objects”*

*There is a word missing or sentence construction not clear.*

**We rephrased the sentence into: “The scANANSE pipeline consists of two components: a package to export and import data from and towards single-cell objects, and a snakemake implementation of ANANSE called anansnake”**

*Please cite R and Python in the text for example in “objects in the programming language Python”*

**We included citations to R and Python**

*“One downside of the GRN modelling of ANANSE is the lack of prediction of repressive TFs.”*

*->This is very interesting, would it be possible to provide an example or a reference to this statement. -> Why is it this case? and how does your approach-combining with motif data-helps? Generally, most TFs are activators and do so by opening the chromatin for transcription.*

**This sentence is based on the “mean rank’ concatenation performed by ANANSE which combines expression data & binding data, and due to the mean rank approach rewards positive correlation (and penalizes repressive anti-correlations). We have added a citation to the original paper after this statement to make clear where this statement arises from. We have added an additional sentence linking to the original ANANSE manuscript after rephrasing the mentioning of the current limitations towards: “One downside of the GRN modelling of ANANSE is the lack of prediction of repressive TFs. This is based on underlying methods of ANANSE (Xu et al., 2021)”**

**We agree with this reviewer’s comment that generally most TFs are activators and open the chromatin. However, in the case where there is interest in repressive factors, the motif-expression correlation analysis incorporated in scANANSE performs a very simple motif accessibility-TF expression correlation analysis, which can identify both positive and negative correlation between a motif and TF expression. It can therefore also predict repressive factors. While it does not use the GRN approach from ANANSE, it does provide a simple method to look at potential repressive factors.**

*“Since ANANSE's assumptions for GRN modelling are not valid for repressive factors, one limitation is the inability to reliably predict repressive TFs.”*

*-> Can you please state these assumptions*

***The method used by ANANSE are described in detail in the original manuscript. However the sentence was rather confusing so we rephrased it into: “*The original ANANSE method was not developed to reliably predict repressive factors (Xu et al., 2021).”**

*I understand that the motif infers TF motif from DEGs and then links them to candidate TFs using expression correlation. Did the authors consider using TF motifs from HT-SELEX experiments in HOCOMOCO or CIS-BP databases instead?*

***scANANSE can utilize any TF motif database. By default, the "gimme.vertebrate.v5.0" motif database from gimmemotifs is utilized, which is based on CIS-BP v1.02, HOCOMOCO v11 and more (doi.org/10.1101/474403). The motif enrichment is performed on are inferred from all ATAC peaks (not the ones limited to DEGs). This motif score is compared with TF expression data from TFs linked to the motif identified.***

*In Table 3, gene names are Italic but not in table1. TFs are proteins and so it is possible to not italicize their symbols in the text and table 5 for example.*

**We fixed the non-italic gene names in table1, and non-italicized the TFs in table 5 and the text.**

*“Although the amount of publicly accessible single-cell datasets is growing, there is an even larger amount of bulk sequencing datasets available”*

*-> This sentence needs to be connected to its main idea.*

**We rephrased the paragraph to connect it better to the main idea:**

**“Furthermore, due to the pseudo-bulk approach, it is possible to compare single-cell cluster gene regulatory networks against networks generated from traditional bulk sequencing data. Although the amount of publicly accessible single-cell datasets is growing, there is an even larger amount of bulk sequencing datasets available. Moreover, the possibility and flexibility of comparing GRNs from multiple sources is another advantage of scANANSE, extra care and validation is still needed when using networks from different data sources. “**

 Finally, I briefly reviewed ANANSE since scANANSE inherits parts of its strengths and weaknesses. A recent benchmark (FIgure 3A in DOI 10.15252/msb.202311627) stated that “enhancer-based network ANANSE showed very poor performance across all cell types”. I believe that this opportunity to address these comments by doing additional benchmarks and identifying the parameter regions/specific use case where scANANSE operates in its optimal regime. I think this conversation can help the community advance on these topics.

***It is true that scANANSE inherits most of the strengths and weaknesses of the original ANANSE algorithm. However, this requires a large amount of work and will be out of the scope of this manuscript. We therefore have not added changes related to this issue in the main text. Nevertheless we wholeheartedly agree that this conversation can help the community and are therefore happy to share our thoughts on the analysis and result. Please note that our comments do not mean to criticize the work of Kamal et al, but to discuss the challenges and possible pitfalls of proper evaluation and comparisons.***

***It is currently very difficult to correctly benchmark GRNs in a thorough and unbiased manner, as also stated by Kamal et al.  In the original ANANSE paper, the GRNs (from ANANSE and other methods) are benchmarked with two different approaches: 1) comparison to known TF-target gene interactions from DoRothEA and 2) comparison to gene expression changes after TF knockdown. This benchmark was performed on fifteen different tissues. Kamal  et al. chose a different benchmark. They train a random forest model to predict gene expression changes from the GRN, and calculate the R2 between predicted expression change and the actual expression change. This is a different metric, on which ANANSE performs poorly.***

***It is important to note that Kamal et al. only tested one ANANSE network, from one specific cell type (in vitro-derived macrophages). In total, they benchmark their GRaNIE method on three cell types (all hematopoietic in origin). This means that it is very hard to draw any solid conclusions on the performance of either method in general.***

***The chosen evaluation metric is interesting and adds to the methods in which GRNs could be evaluated. However, it also has some possible pitfalls. First, only genes which are present in the GRN are included in the analysis. This means that GRN size influences the outcome of the result.  For instance, the GRaNIE naïve macrophage network contains just 2663 TF-gene interactions and consequently, only 9% of the ~7,000 differential genes are evaluated by the R2 metric. If different GRNs have different target genes, this means that not the same expression changes are compared. Different genes (functional categories, promoter sequence content, etc.) may have varying degrees of predictability. Second, the R2 metric is influenced by large outliers. If a few genes with high fold changes have good predictions, this will skew the results.***

***The method of inferring GRNs is very different between ANANSE and GRaNIE. ANANSE uses a single set (or the average of replicates) of expression data and enhancer activity (ATAC-seq and/or H3K27ac) from one cell type to predict the GRN of that specific cell type. However, GRaNIE incorporates co-varying gene-enhancer pairs. This means that GRaNIE needs a large number of input samples to work. All the examples in Kamal et al. use at least ~30-40 samples. Importantly, this will also skew the inferred interactions to genes that change in expression between these samples. In the example of the naïve macrophages, the samples are macrophages from different donors. The regulatory interactions will then be based on genes that change in expression due to variation (i.e. eQTLs) or due to cellular response to growth conditions etc. such as stress. This is visible in the transcription factors with a high number of targets, which include AP1 (Fos) and NFKb (Rel), which are involved in stress response. One other notable set of TFs in the GRaNIE GRN contains factors such as PURA, MBD2, ZBT14, KLF13, E2F7 that have GC-rich motifs and hint towards a promoter/CpG-island based enrichment of the method. It is unlikely that these are relevant.In contrast, ANANSE has a focus on TFs that define or determine cell identity and contains many interactions for genes such as SPI1 and CEBPB. These TFs are necessary and sufficient to differentiate pluripotent cells to macrophages. The GRaNIE network does not capture this and contains only 9 interactions for these factors.***

***Finally, the scopes of the network are very different. The GRaNIE macrophage network consists of 2,663 interactions, the ANANSE network contains all TF to target gene interactions. It is likely that many of the interactions in the GRaNIE network are true positives (i.e. high precision), but many more will be missed. Networks from ANANSE, on the other hand, will likely be much more noisy but can serve as a source to model cellular decisions. This will identify relevant TFs as demonstrated benchmarked in the original ANANSE paper.***

***In short, the method, scope and applicability of the two methods is different. Unsurprisingly, both chose relevant but different benchmarks to evaluate the respective method.***

- *Is the rationale for developing the new software tool clearly explained?*

*Yes*

- *Is the description of the software tool technically sound?*

*Yes*

- *Are sufficient details of the code, methods and analysis (if applicable) provided to allow replication of the software development and its use by others?*

*Yes*

- *Is sufficient information provided to allow interpretation of the expected output datasets and any results generated using the tool?*

*Yes*

- *Are the conclusions about the tool and its performance adequately supported by the findings presented in the article?*

*Yes*

**References**

 1. Kamal A, Arnold C, Claringbould A, Moussa R, et al.: GRaNIE and GRaNPA: inference and evaluation of enhancer-mediated gene regulatory networks. *Mol Syst Biol*. 2023; **19** (6): e11627 PubMed Abstract | Publisher Full Text

# Reviewer report: Reviewer response for version 1

**DOI:** 10.5256/f1000research.143297.r175202

**Published:** 2023-06-19

**Authors:** Kenji Kamimoto

**Affiliations:** 1 Department of Developmental Biology, Washington University School of Medicine in St. Louis, St. Louis, MO, USA

The authors have developed scANANSE, a pipeline for gene network analysis and transcription factor binding motif analysis. scANANSE is a software tool that facilitates the creation of cell type-specific networks from gene expression data and ATAC-seq data. scANANSE employs pseudo bulk data, enabling comparisons with algorithms that rely on bulk data.

 scANANSE serves as an extension package of ANANSE, incorporating additional features such as data conversion from single cell data and the ability to return network analysis results to single cell data. Although data processing and manipulation are often overlooked aspects in general, they hold significant importance. This software offers the advantage of easily performing such operations, which enhances its practicality. The manuscript effectively conveys the purpose, adaptations, and limitations of scANANSE, while also providing clear examples of code usage. The overall organization is well-executed.

 I have no major comments or requests to add. I will provide some minor feedback regarding specific findings from utilizing the software.

 1. I got error when importing SeuratDisk. The SeuratDisk may not be included in the installation.

 Also, it seems SeuratDisk is not available from CRAN for R version 4. I installed SeuratDisk from GitHub.

 It may be helpful to add this process in installation.

 ""

 Attaching SeuratObject

 Error in library(SeuratDisk) : there is no package called 'SeuratDisk'

 ""

 2. At page8, the file name of Seurat object may include typo.

 The file name in R script is "preprocessed_PDMC.Rds". It should be "preprocessed_PBMC.Rds"

Are the conclusions about the tool and its performance adequately supported by the findings presented in the article?

Yes

Is the rationale for developing the new software tool clearly explained?

Yes

Is the description of the software tool technically sound?

Yes

Are sufficient details of the code, methods and analysis (if applicable) provided to allow replication of the software development and its use by others?

Yes

Is sufficient information provided to allow interpretation of the expected output datasets and any results generated using the tool?

Yes

Reviewer Expertise:

Systems biology, Developmental Biology, Single cell genomics

I confirm that I have read this submission and believe that I have an appropriate level of expertise to confirm that it is of an acceptable scientific standard.

# Response

**Published:** 2023-11-24

**Authors:** Jos Smits

**Affiliations:** Radboud University Nijmegen, The Netherlands

**Dear Dr. Kenji Kamimoto,**

**Thank you for providing suggestions to further improve our manuscript. We have revised the manuscript accordingly. A point-by-point response can be found bellow. Next to incorporating the suggested changes we have also updated AnanseSeurat to be compatible with the recently released Seurat V5.**

*The authors have developed scANANSE, a pipeline for gene network analysis and transcription factor binding motif analysis. scANANSE is a software tool that facilitates the creation of cell type-specific networks from gene expression data and ATAC-seq data. scANANSE employs pseudo bulk data, enabling comparisons with algorithms that rely on bulk data.*

*scANANSE serves as an extension package of ANANSE, incorporating additional features such as data conversion from single cell data and the ability to return network analysis results to single cell data. Although data processing and manipulation are often overlooked aspects in general, they hold significant importance. This software offers the advantage of easily performing such operations, which enhances its practicality. The manuscript effectively conveys the purpose, adaptations, and limitations of scANANSE, while also providing clear examples of code usage. The overall organization is well-executed.*

*I have no major comments or requests to add. I will provide some minor feedback regarding specific findings from utilizing the software.*

*1. I got error when importing SeuratDisk. The SeuratDisk may not be included in the installation.*

*Also, it seems SeuratDisk is not available from CRAN for R version 4. I installed SeuratDisk from GitHub.*

*It may be helpful to add this process in installation.*

*""*

*Attaching SeuratObject*

*Error in library(SeuratDisk) : there is no package called 'SeuratDisk'*

*""*

**We added instructions to install SeuratDisk from Github.**

*2. At page8, the file name of Seurat object may include typo.*

*The file name in R script is "preprocessed_PDMC.Rds". It should be "preprocessed_PBMC.Rds"*

**We fixed the typo**

- *Is the rationale for developing the new software tool clearly explained?*

*Yes*

- *Is the description of the software tool technically sound?*

*Yes*

- *Are sufficient details of the code, methods and analysis (if applicable) provided to allow replication of the software development and its use by others?*

*Yes*

- *Is sufficient information provided to allow interpretation of the expected output datasets and any results generated using the tool?*

*Yes*

- *Are the conclusions about the tool and its performance adequately supported by the findings presented in the article?*

*Yes*
